# Comparison of progesterone assay by chemiluminescence or radioimmunoassay for clinical decision-making in canine reproduction

**DOI:** 10.4102/jsava.v90i0.1730

**Published:** 2019-10-22

**Authors:** Johan O. Nöthling, Kurt G.M. De Cramer

**Affiliations:** 1 Department of Production Animal Studies, Section of Reproduction, Faculty of Veterinary Science, Onderstepoort, South Africa; 2 Rant en Dal Animal Hospital, Mogale City, South Africa

**Keywords:** progesterone, bitch, radioimmunoassay, Coat-A-Count, Immulite, luteinizing hormone, LH

## Abstract

The Coat-A-Count® radioimmunoassay has been long and widely used to determine the concentration of progesterone in serum or plasma of bitches (progRIA), but was discontinued in 2014. The Immulite® 1000 LKPG1 chemiluminescence immunoassay has gained prominence since 2003 to determine the concentration of progesterone in serum of bitches, but the assay changed in 2012 (Immulite® 1000 LKPW1). This study assessed the feasibility of using Immulite® 1000 LKPW1 (progImm) to estimate the time of clinically relevant events during oestrus and compared progRIA and progImm 2 and 3 days after the first or only day of the luteinizing hormone surge (LH1). ProgImm first exceeded 5.1 nmol/L on the same day that progRIA first exceeded 6 nmol/L, a proxy for the occurrence of the LH surge, or the day before in 28 of 31 (90%) of oestrous periods. ProgImm first exceeded 13.6 nmol/L on the same day that progRIA first exceeded 16 nmol/L (a proxy for the day of ovulation) or the day before in 34 of 35 (97%) oestrous periods. ProgImm first exceeded 5.4 nmol/L on LH1 or the day before in 24 of 25 (95%) of oestrous periods. The median of progImm 2 days after LH1 was 1.2 nmol/L lower than the 10.7 nmol/L of progRIA (*p* = 0.001). The mean of progImm 3 days after LH1 was 2.2 nmol/L lower than the 19.0 nmol/L of progRIA (*p* < 0.001). In conclusion, the days on which progImm first exceeded 5.1 nmol/L, 13.6 nmol/L and 5.4 nmol/L effectively estimate the days on which progRIA reached 6 nmol/L or 16 nmol/L or LH1.

## Background

The concentration of progesterone (P4) in blood plasma (plasma) or serum of bitches is determined and used for various clinical decisions. Anoestrus is characterised by a (P4) ≤ 1.3 nmol/L (Jeffcoate 1993) or ≤ 1.6 nmol/L (Concannon 2011). The first day of the oestrous cycle on which (P4) exceeds 6 nmol/L occurs 6 days before the day with the highest expected fertility using frozen-thawed spermatozoa (Steckler, Nöthling & Harper 2013). The luteinizing hormone (LH) surge is estimated to start when (P4) reaches 3 nmol/L (Concannon 2011). The LH peak is estimated to occur when (P4) reaches 5.1 nmol/L (Concannon, Hansel & Visek 1975) or 8.3 nmol/L (Concannon, Hansel & McEntee 1977) or 9.8 nmol/L (Bergeron et al. 2013). Ovulation occurred in 26 of 39 bitches once (P4) reached 15.9 nmol/L and in 55 of 59 within 1 day thereof (Fontbonne 2008), whereas the mean (P4) at the time of ovulation was 19.4 nmol/L (standard deviation [s.d.] 3.72) according to Gropetti et al. (2015).

The Coat-A-Count ^125^I radioimmunoassay (RIA) for progesterone by Siemens has commonly been used in research and practice to determine (P4) in the serum or plasma of bitches for decades (Gerstenberg & Nöthling 1995; Kutzler et al. 2003; Luz et al. 2006; Okkens et al. 2001; Reimers et al. 1991; Steckler et al. 2013), but was due to stop during the last quarter of 2014. Immulite, a chemiluminescent immunoassay, became popular for measuring (P4) in the serum of bitches (Chapwanya et al. 2008; Kutzler et al. 2003; Rota et al. 2015; Schmicke et al. 2016; Volkmann 2006).

Nöthling and De Cramer (2018) compared the (P4) measured with the Immulite 1000 LKPW1 assay (progImm) in serum to those measured with the Coat-A-Count radioimmunoassay (progRIA) in blood plasma collected at the same time from the same bitches. On average, (P4) obtained with progImm was 85% that of ProgRIA, with a wide scatter around the mean (95% confidence interval [CI], 58% – 112%). Eighty-eight percent of the progImms were lower than the progRIAs.

The different results yielded by these two assays suggested a need to determine the accuracy of using the Immulite LKPW1 assay instead of the Coat-A-Count RIA to identify clinically important stages of the oestrous cycle in bitches.

## Objective

The aim of this study was to determine five clinically important effects. They were the differences in (1) the dates on which the concentration of progesterone first exceeds 6 nmol/L (a proxy for the occurrence of the LH surge) or (2) 16 nmol/L (a proxy for the day of ovulation), (3) the concentrations of progesterone on the first or only day of the LH surge (LH1) and (4) the concentrations of progesterone 2 and 3 days after LH1 (when ovulation occurs) and (5) the number of days between LH1 and the date on which it is expected to occur based on the concentration of progesterone.

## Materials and methods

### Collection of specimens and hormone analyses

Vaginal cytology and vaginal speculum examination were used to identify pro-oestrus and oestrus during one oestrus cycle in bitches (*n* = 43; 18 English bulldog, 25 Boerboel). Two vials of blood (BD VAC PLAIN 10 mL glass tube and BD Vacutainer® 170 IU lithium heparin 10 mL tube; BD Plymouth, United Kingdom) were collected by jugular venepuncture immediately after each other, around 08:00 in the morning, once every 24 hours or 48 hours during late pro-oestrus and oestrus from each bitch. The serum and plasma samples were harvested from the 194 pairs of blood samples so obtained and stored in Plastpro cryovials with a screw top and a silicone washer (Plastpro Pty Ltd, Johannesburg). Each cryovial was labelled with a unique 4-digit random number only and frozen at 18°C until assayed. All RIA assays were performed within 1–3 months after the plasma samples were collected. Based on the progRIAs so obtained, 110 serum samples were selected for the Immulite assay, which were done in 3 batches over a 2-day period, 4–8 months after the serum samples were collected. The 110 progImms so obtained were used to compare to the progRIAs as described in a related study (Nöthling & De Cramer 2018). Eighty-nine of these 110 progImms were included in the current study. Based on the stage of the oestrous cycle, the bitches were on when the serum samples were collected; the remaining 105 Immulite assays were performed 19–23 months after the serum samples were collected. One hundred of these 105 were done in a single batch on the same day and the remaining 5 in another batch on another day.

The concentration of progesterone in plasma samples was determined with RIA (Coat-A-Count® radioimmunoassay; Siemens Health Care Diagnostics Inc., Los Angeles, CA, United States) and in serum samples with Immulite (Immulite® 1000 Catalogue number LKPW1; Siemens Medical Solutions Diagnostics, Los Angeles, CA, United States). All determinations were done in duplicate. The mean of the two replicate concentrations determined with RIA and Immulite is referred to as progRIA and progImm.

The intra-assay coefficient of variation (CV) was determined for each pair of replicate plasma and serum samples. The mean intra-assay CVs for RIA are reported for the 22 plasma samples with progRIAs between 2.0 nmol/L and 4.0 nmol/L (low concentrations), the 19 with progRIAs between 13.0 nmol/L and 14.0 nmol/L (moderate concentrations) and the 19 with progRIAs between 22.0 nmol/L and 23.0 nmol/L (high concentrations). The mean intra-assay CVs for Immulite are also reported for low, moderate and high concentrations using the 22, 19 and 19 serum samples derived from the same blood samples used to determine the intra-assay CVs for RIA.

Two plasma samples of which the first progRIA was between 2 nmol/L and 4 nmol/L (low concentration), two of which was between 13 nmol/L and 14 nmol/L (moderate concentration) and two of which was between 22 nmol/L and 23 nmol/L (high concentration) were assayed 6 times on different dates to determine the mean inter-assay CV for RIA at each of these concentration buckets. The six serum samples derived from the same blood samples as the six plasma samples were each assayed three times in different batches with Immulite to determine the inter-assay CVs for Immulite.

The Witness® LH test (Synbiotics Europe, Lyon, France) was used on 159 of the serum samples to determine LH surges in 43 bitches.

Following the above, progRIA and progImm were both available from 32 bitches on the day that progRIA first exceeded 6 nmol/L as well as the day before, 35 on the day on which progRIA first exceeded 16 nmol/L as well as the day before, 27 on the first or only day that the concentration of LH in the serum was elevated (LH1), 27 on 2 days after and 25 on 3 days after LH1.

### Data analysis

Previously, it was shown that progImm commonly underestimates progRIA (Nöthling & De Cramer 2018). Here we determined whether progRIA was higher than progImm (1) on the day on which progRIA first exceeded 6 nmol/L, (2) on the day on which progRIA first exceeded 16 nmol/L, (3) on LH1, (4) 2 days after LH1 and (5) 3 days after LH1. A 1-tailed paired *t*-test or Wilcoxon’s signed rank test or the sign test was used to compare (P4) measured by the two methods.

The number of days from progRIA having first exceeded 6 nmol/L until progImm having done so was determined by direct inspection of the dataset. Having shown that progImm is expected to be 85% that of progRIA throughout a range in progRIA from 2 nmol/L to 25 nmol/L (18), the same was done to determine the number of days from progRIA having first exceeded 6 nmol/L until progImm first exceeded 5.1 nmol/L (5.1 = 0.85 × 6). We compared the proportions of bitches in which progImm first exceeded 6 nmol/L or 5.1 nmol/L on the same day that progRIA first exceeded 6 nmol/L. We also compared the proportions of bitches in which progImm first exceeded 6 nmol/L or 5.1 nmol/L during a 2-day period that included the day on which progRIA first exceeded 6 nmol/L.

The above was repeated with respect to the interval between progRIA having first exceeded 16 nmol/L until progImm first exceeded 16 nmol/L or its expected value of 13.6 nmol/L.

The number of days between LH1 and the date on which it was expected to occur based on progRIA or progImm was determined by direct inspection of the data.

Using Fisher’s exact test, we compared the proportions of bitches in which LH1 occurred on the expected day based on progRIA or progImm, respectively, or within 2 days thereof.

Data analysis was performed using Stata 14 (Stata Corp College Station, TX, United States).

### Ethical considerations

The project was approved by the Animal Ethics Committee of the Faculty of Veterinary Science of the University (Projects v071-13, v010-14).

## Results

The intra-assay CVs for low, moderate and high concentrations of progesterone were 10.6%, 5.7% and 4.4% for RIA and 7.7%, 5.9% and 4.1% for Immulite. The inter-assay CVs for low, moderate and high concentrations of progesterone were 13.1%, 7.2% and 8.3% for RIA and 6.8%, 7.8% and 12.9% for Immulite.

ProgImm was lower than progRIA on the day that progRIA first exceeded 6 nmol/L or 16 nmol/L, as well as on LH1 and 2 or 3 days thereafter (Table 1).

**TABLE 1:** Concentrations of progesterone measured with Coat-A-Count® radioimmunoassay and Immulite® 1000 LKPW1 at important times during oestrus in bitches.

Stage during the oestrous cycle	Minimum	Twenty-fifth percentile	Median	Seventy-fifth percentile	Maximum	Mean	s.d.	*n*	*p*
**On the day that RIA exceeded 6 nmol/L**									
RIA	6.2	7.0	8.1	9.3	10.5	8.11	1.38	32	0.0354*
Immulite	4.1	6.2	7.1	8.3	14.2	7.65	2.09	32	-
**On the day that RIA exceeded 16 nmol/L**									
RIA	16.2	20.2	23.0	28.3	52.6	24.67	7.43	35	< 0.001**
Immulite	13.5	15.7	20.0	24.8	50.9	21.43	7.87	35	-
**On the first or only day of the LH surge**									
RIA	1.5	4.9	6.2	7.4	10.0	6.06	2.09	27	0.007***
Immulite	1.1	4.0	5.6	7.0	9.1	5.41	1.96	27	-
**Two days after the first or only day of the LH surge**									
RIA	5.0	9.5	10.7	12.5	22.2	11.58	3.71	27	0.001
Immulite	3.8	8.3	9.5	11.7	18.8	10.08	2.96	27	-
**Three days after the first or only day of the LH surge**									
RIA	9.8	15.2	17.8	23.0	28.5	18.95	5.30	25	< 0.001**,***
Immulite	9.9	13.5	15.1	20.2	28.8	16.73	4.68	25	-

s.d., standard deviation; RIA, radioimmunoassay; LH, Luteinizing hormone.

* , One-tailed sign test, testing whether the median concentration of progesterone measured with Immulite is lower than that for radioimmunoassay.

** , One-tailed signed rank test, testing whether the median concentration of progesterone measured with Immulite is lower than that for radioimmunoassay.

*** , One-tailed paired t-test test, testing whether the mean concentration of progesterone measured with Immulite is lower than that for radioimmunoassay.

### Using Immulite to identify the day on which progRIA first exceeded 6 nmol/L

**FIGURE 1:**
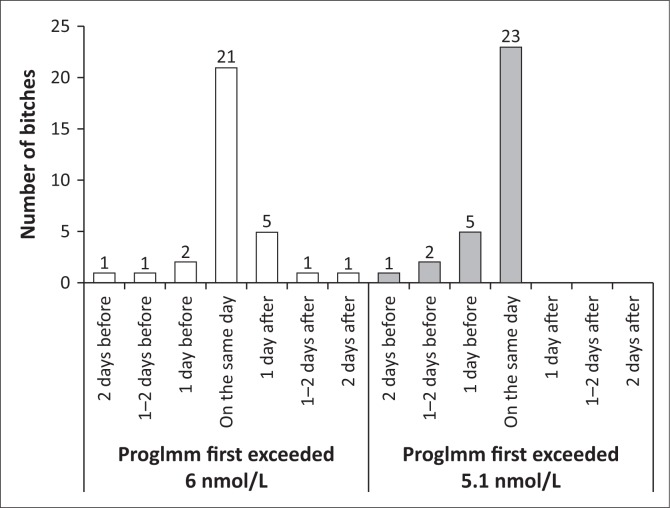
Days – relative to the day on which the concentration of progesterone measured with Coat-A-Count® radioimmunoassay in the plasma of bitches first exceeded 6 nmol/L – on which the concentration measured in their serum with Immulite® 1000 LKPW1 (ProgImm) first exceeded 6 nmol/L or its predicted value of 5.1 nmol/L.

ProgImm, respectively, first exceeded 6 nmol/L or 5.1 nmol/L on the same day that progRIA first exceeded 6 nmol/L in 21 of 32 bitches (66%, 95% CI, 47% – 81%) or 23 of 31 bitches (74%, 95% CI, 55% – 88%), *p* = 0.58 (Figure 1).

The interval range between progImm first exceeding 6 nmol/L or 5.1 nmol/L and progRIA first exceeding 6 nmol/L was 4 and 2 days, respectively (Figure 1).

ProgImm first exceeded 6 nmol/L on the day that progRIA did or the day thereafter in 26 of 32 bitches (82%, 95% CI, 64% – 93%), which was similar to the 28 of 31 bitches (90%, 95% CI, 74% – 98%) in which progImm first exceeded 5.1 nmol/L on the day that progRIA first exceeded 6 nmol/L or the day before (*p* = 0.48).

### Using Immulite to identify the day on which the concentration of progesterone measured with radioimmunoassay first exceeded 16 nmol/L

**FIGURE 2:**
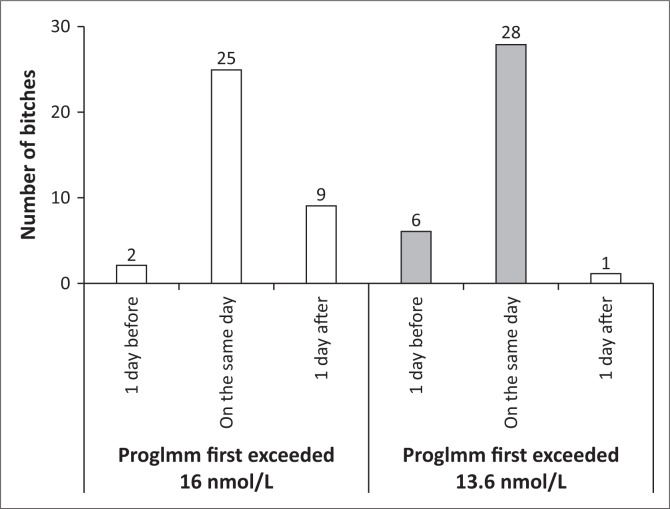
Days – relative to the day on which the concentration of progesterone measured with Coat-A-Count® radioimmunoassay in the plasma of bitches first exceeded 16 nmol/L – on which the concentration measured in their serum with Immulite® 1000 LKPW1 (ProgImm) first exceeded 16 nmol/L or its predicted value of 13.6 nmol/L.

ProgImm, respectively, first exceeded 16 nmol/L or 13.6 nmol/L on the day that progRIA first exceeded 16 nmol/L in 25 of 36 bitches (69%, 95% CI, 52% – 84%) or 28 of 35 bitches (80%, 95% CI, 63% – 92%), *p* = 0.42 (Figure 2).

The range in interval between progImm first exceeding 16 nmol/L or 13.6 nmol/L and progRIA first exceeding 16 nmol/L was 2 days in each case (Figure 2).

Figure 2 shows that the day on which progImm first exceeded 16 nmol/L coincided with the day on which progRIA first exceeded 16 nmol/L or the day following that in 34 (94%) of 36 bitches (95% CI, 81% – 99%). This proportion was similar to the 34 (97%) of 35 bitches (95% CI, 85% – 100%) in which progImm first exceeded 13.6 nmol/L on the day on which progRIA first exceeded 16 nmol/L or the day before (*p* = 1.0).

### Identifying the luteinizing hormone surge using the concentration of progesterone

The LH surge was detected in 32 bitches. Its duration was determined in 25 bitches because it was confirmed that the first day of the surge was preceded and its last day followed by days without elevated concentrations of LH. The LH surge lasted one day in 18 bitches, two days in 5 and three days in 2. In 7 of the 32 bitches, the duration of the LH surge could not be determined. In these 7 bitches, the LH surge lasted at least one day in 3, at least 2 days in 3 and at least 4 days in one.

**FIGURE 3:**
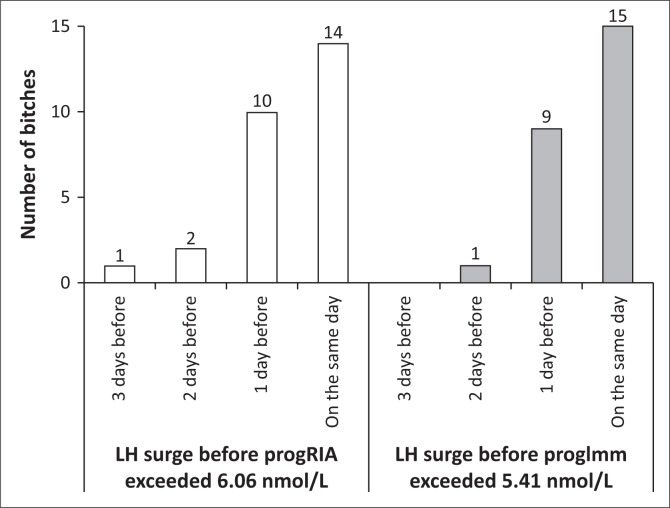
Days, relative to the first or only day of the LH surge, on which the concentration of progesterone measured with Coat-A-Count® radioimmunoassay in the plasma (progRIA) or with Immulite® 1000 LKPW1 in the serum (progImm) of bitches, respectively, first exceeded 6.06 nmol/L or 5.41 nmol/L (which were their mean concentrations on the day of the LH surge).

The mean of progRIA on LH1 was 6.06 nmol/L, which was higher than the 5.41 nmol/L for progImm (*p* = 0.007) (Table 1). The luteinizing hormone surge occurred on the day that progRIA first exceeded 6.06 nmol/L in 14 of 27 bitches (52%, 95% CI, 32% – 71%) or ProgImm first exceeded 5.41 nmol/L in 15 of 25 bitches (60%, 95% CI, 39% – 79%), *p* = 0.59 (Figure 3).

In 24 of 27 bitches (89%, 95% CI, 71% – 98%) and in 24 of 25 bitches (96%, 95% CI, 80% – 100%) LH1 occurred on the day or the day before progRIA first exceeded 6.06 nmol/L or progImm first exceeded 5.41 nmol/L (*p* = 0.61) (Figure 3).

The range in interval between progRIA first exceeding 6.06 nmol/L or progImm first exceeding 5.41 nmol/L and LH1 was 3 and 2 days, respectively (Figure 3).

### ProgRIA and progImm 2 and 3 days after the first or only day of the luteinizing hormone surge

The median of progImm 2 days after LH1 was 1.2 nmol/L lower than the 10.7 nmol/L of progRIA (*p* = 0.001) (Table 1). The mean of progImm 3 days after LH1 was 2.2 nmol/L lower than the 19.0 nmol/L of progRIA (*p* ≤ 0.001) (Table 1).

## Discussion

### Using progImm to estimate the day on which progRIA would first have exceeded 6 nmol/L

Steckler et al. (2013) showed that the fertility of frozen-thawed spermatozoa is higher when bitches are inseminated six days after progRIA first exceeded 6 nmol/L than five or seven days thereafter. The low expected probability and its wide 95% CI found in the current study show that neither a progImm of 5.1 nmol/L nor one of 6 nmol/L is effective in estimating the day that progRIA would first exceed 6 nmol/L. The high probability of a progImm of 5.1 nmol/L correctly estimating the day on which progRIA would first exceed 6 nmol/L or the day before renders it useful to predict the optimal time to inseminate bitches with frozen-thawed spermatozoa: there is a 90% probability (95% CI, 74% – 98%) that a bitch receiving her first insemination 6 days after progImm has first exceeded 5.1 nmol/L will be inseminated five or six days after progRIA would first have exceeded 6 nmol/L. Inseminating her again 24 hours later would, again with a 90% probability of success, ensure that the second insemination occurs either six or seven days after progRIA first exceeded 6 nmol/L. Such an insemination schedule will ensure insemination on the most fertile day together with one day before or after, which have been shown to result in more conceptuses in a bitch than would be the case if she is only inseminated on the most fertile day (Steckler et al. 2013).

### Using progImm to estimate the day on which progRIA would first have exceeded 16 nmol/L

The day on which the concentration of progesterone in plasma reaches 16 nmol/L provides a good estimate of the time of ovulation (Fontbonne 2008). Okkens et al. (2001) found that a single mating one day after the concentration of progesterone measured with the Coat-A-Count RIA first exceeds 16 nmol/L yields optimal fertility. The current study shows that neither the day on which progImm first exceeds 16 nmol/L nor the day on which it first exceeds 13.6 nmol/L is effective in estimating the day on which progRIA would first exceed 16 nmol/L. The day on which progImm first exceeds 13.6 nmol/L provides a very good estimate of the day on which progRIA would first exceed 16 nmol/L or the day before, with a 97% probability of being correct and a 95% CI of 85% – 100%. The day on which progImm first exceeds 13.6 nmol/L therefore provides a suitable means of estimating the time of ovulation (Fontbonne 2008) and the time of breeding (Okkens et al. 2001).

### Using progImm to estimate the first or only day of the luteinizing hormone surge

Nishiyama et al. (1999) showed that the day of the LH surge identified with the LH assay kit, now known as Witness, corresponds well with the day identified by measuring the concentration of LH with a radioimmunoassay. Tsumagari et al. (2003) inseminated bitches five and seven days after the LH surge as determined using the same LH kit and achieved optimal fertility. Among studies in which progesterone in plasma or serum was assayed once a day, the mean progRIA of 6.06 nmol/L (s.d. 2.09) on the first or only day of the LH surge in the current study is similar (*p* > 0.05) to the 5.088 nmol/L (SE 0.64) and the 7.0 nmol/L (SE 1.69) that Concannon et al. (1975) and Fontbonne (2008) reported on the day when the concentration of LH in the plasma was highest, but differs (*p* < 0.05) from the 9.76 nmol/L (s.d. 3.37) reported by Bergeron et al. (2013). The mean concentration of progesterone on the first or only day of the LH surge in the current study differs from the 8.14 nmol/L (SE 0.95) at the time of the LH peak in a study where plasma was collected hourly eight times (Concannon et al. 1977). In the current study, 18 of 32 LH surges lasted 1 day only and the remainder 2 days or more. This finding supports that of Concannon et al. (1975) that showed that 10 of 20 LH surges lasted one day and the remainder 2 or more.

The current study shows that the day on which progRIA first exceeded 6.06 nmol/L and the day on which progImm first exceeded 5.41 nmol/L are ineffective in estimating the first or only day of the LH surge. Both are effective in estimating the first or only day of the LH surge or the day preceding that, with 89% and 96% probabilities of being correct. The latter probability has a 95% CI of 80% – 100%, suggesting that the day on which progImm first exceeds 5.41 nmol/L is expected to correctly indicate the first or only day of the LH surge or the day preceding it in at least 80% of oestrous periods. In the clinical setting, this means that inseminating a bitch with frozen-thawed semen six days after progImm first exceeds 5.1 nmol/L will, in at least 80% of bitches, occur five or six days after the first or only day of the LH surge. Following this with a second insemination 1 day later will, in at least 80% of bitches, result in the two inseminations occurring in the optimal period (Tsumagari et al. 2003).

### The agreement between progImm and progRIA 2 and 3 days after the first or only day of the luteinizing hormone surge

Ovulation occurs 2–3 days after the LH peak (Concannon et al. 1977; Fontbonne 2008; Phemister et al. 1973). Although significantly lower than progRIA, various summary statistics of progImm were numerically close to those of progRIA 2 and 3 days after the first or only day of the LH surge. The mean progImm of 10.1 nmol/L (s.d. 2.96) 2 days after the LH surge was close to the 10.8 nmol/L (s.d. 2.86) that Schmicke et al. (2016) found at the time of ovulation using the same assay as the one used in the current study.

## Conclusion

Using the Immulite® 1000 LKPW1 chemiluminescence immunoassay instead of the Coat-A-Count radioimmunoassay to determine the concentration of progesterone in the serum or plasma of bitches required a change in the target concentrations associated with critical events during oestrus, as well as a change in the interpretation of the temporal relationship between reaching the respective target concentrations with the two assays.

## References

[CIT0001] Bergeron L.H., Nykamp S.G., Brisson B.A., Madan P. & Gartley C.J., 2013, ‘An evaluation of B-mode and color Doppler ultrasonography for detecting periovulatory events in the bitch’, *Theriogenology* 79(2), 274–283. 10.1016/j.theriogenology.2012.08.016 23174775

[CIT0002] Chapwanya A., Clegg T., Stanley P. & Vaughan L., 2008, ‘Comparison of the Immulite and RIA assay methods for measuring peripheral blood progesterone levels in Greyhound bitches’, *Theriogenology* 70(5), 795–799. 10.1016/j.theriogenology.2008.05.047 18579195

[CIT0003] Concannon P., Hansel W. & McEntee K., 1977, ‘Changes in LH, progesterone and sexual behaviour associated with preovulatory luteinization in the bitch’, *Biology of Reproduction* 17(4), 604–613. 10.1095/biolreprod17.4.604 562686

[CIT0004] Concannon P.W., 2011, ‘Reproductive cycles of the domestic bitch’, *Animal Reproduction Science* 124(3–4), 200–210. 10.1016/j.anireprosci.2010.08.028 21055888

[CIT0005] Concannon P.W., Hansel W. & Visek W.J., 1975, ‘The ovarian cycle of the bitch: Plasma estrogen, LH and progesterone’, *Biology of Reproduction* 13(1), 112–121. 10.1095/biolreprod13.1.112 1222178

[CIT0006] Fontbonne A., 2008, ‘In vivo ovulation, oocyte maturation and fertilisation in the bitch’, PhD thesis, Life Sciences, Paris Institute of Technology for Food, Life and Environmental Sciences (Agro Paris Tech).

[CIT0007] Gerstenberg C. & Nöthling J.O., 1995, ‘The effects of metergoline combined with PGF2α, treatment on luteal function and gestation in pregnant bitches’, *Theriogenology* 44(5), 649–659. 10.1016/0093-691X(95)00245-4 16727763

[CIT0008] Gropetti D., Aralla M., Bronzo V., Bosi G., Pecile A. & Arrighi S., 2015, ‘Periovulatory time in the bitch: What’s new to know? Comparison between ovarian histology and clinical features’, *Animal Reproduction Science* 152, 108–116. 10.1016/j.anireprosci.2014.11.008 25510561

[CIT0009] Jeffcoate I.A., 1993, ‘Endocrinology of anoestrous bitches’, *Journal of Reproduction and Fertility* 47, 69–76. 8229987

[CIT0010] Kutzler M.A., Mohammed H.O., Lamb S.V. & Meyers-Wallen V.N., 2003, ‘Accuracy of canine parturition date prediction from the initial rise in preovulatory progesterone concentration’, *Theriogenology* 60(6), 1187–1196. 10.1016/S0093-691X(03)00109-2 12935856

[CIT0011] Luz M.R., Bertan C.M., Binelli M. & Lopes M.D., 2006, ‘Plasma concentrations of 13,14-dihydro-15-keto prostaglandin F2-alpha (PGFM), progesterone and estradiol in pregnant and nonpregnant diestrus cross-bred bitches’, *Theriogenology* 66(6–7), 1436–1441. 10.1016/j.theriogenology.2006.01.036 16464490

[CIT0012] Nishiyama T., Kinugasa T., Kimura T., Watanabe G., Taya K., Tsumagari S. et al., 1999, ‘Determination of optimal time for mating by artificial insemination with chilled semen using luteinizing hormone surge as an indicator in beagles’, *Journal of the American Animal Hospital Association* 35(4), 348–352. 10.5326/15473317-35-4-348 10416781

[CIT0013] Nöthling J.O. & De Cramer K.G.M., 2018, ‘Comparing the values of progesterone in the blood of bitches as measured with a chemiluminescence immunoassay and a radioimmunoassay’, *Reproduction in Domestic Animals* 53(5), 1136–1141. 10.1111/rda.13216 29938844

[CIT0014] Okkens A.C., Teunissen J.M., Van Osch W., Van Den Brom W.E., Dieleman S.J. & Kooistra H.S., 2001, ‘Influence of litter size and breed on the duration of gestation in dogs’, *Journal of Reproduction and Fertility* suppl. 57, 193–197. 11787149

[CIT0015] Phemister R.D., Holst P.A., Spáno J.S. & Hopwood M.L., 1973, ‘Time of ovulation in the beagle bitch’, *Biology of Reproduction* 8(1), 74–82. 10.1093/biolreprod/8.1.74 4734806

[CIT0016] Reimers T.J., Lamb S.V., Bartlett S.A., Matamoros R.A., Cowan R.G. & Engle J.S., 1991, ‘Effects of hemolysis and storage on quantification of hormones in blood samples from dogs, cattle, and horses’, *American Journal of Veterinary Research* 52(7), 1075–1080. 1892262

[CIT0017] Rota A., Charles C., Cucuzza A.S. & Pregel P., 2015, ‘Diagnostic efficiency of a single progesterone determination to assess full-term pregnancy in the bitch’, *Reproduction in Domestic Animals* 50, 1028–1031. 10.1111/rda.12631 26510871

[CIT0018] Schmicke M., Urhausen C., Wolf K., Schmidt S. & Günzel-Apel A., 2016, ‘Evaluation der mittels Chemilumineszenztest gemessenen Blut-Progesteronkonzentration bei der Hündin am Tag der Ovulation’ [Evaluation of the blood progesterone concentration in the bitch measured by chemiluminescence immunoassay at the day of ovulation], *Tierarztliche Praxis Ausgabe K: Kleintiere – Heimtiere* 44(5), 317–322. 10.15654/TPK-150364 27277935

[CIT0019] Steckler D., Nöthling J.O. & Harper C., 2013, ‘Prediction of the optimal time for insemination using frozen-thawed semen in a multi-sire insemination trial in bitches’, *Animal Reproduction Science* 142, 191–197. 10.1016/j.anireprosci.2013.09.013 24128644

[CIT0020] Tsumagari S., Ichikawa Y., Toriumi H., Ishihama K., Morita M., Kanamaki M. et al., 2003, ‘Optimal timing for canine artificial insemination with frozen semen and parentage testing by microsatellite markers for superfecundency’, *Journal of Veterinary Medical Science* 65(9), 1003–1005. 10.1292/jvms.65.1003 14532694

[CIT0021] Volkmann D.H., 2006, ‘The effects of storage time and temperature and anticoagulant on laboratory measurements of canine blood progesterone concentrations’, *Theriogenology* 66(6–7), 1583–1586. 10.1016/j.theriogenology.2006.01.024 16480764

